# Medication-related Osteonecrosis of the Jaw: A Review

**DOI:** 10.7759/cureus.6944

**Published:** 2020-02-10

**Authors:** Nouf A AlDhalaan, Asma BaQais, Ahmad Al-Omar

**Affiliations:** 1 Dentistry, King Saud University, Riyadh, SAU; 2 Surgery, King Saud University, Riyadh, SAU

**Keywords:** necrosis, bisphosphonates, denosumab, bronj

## Abstract

Medication-related osteonecrosis of the jaw (MRONJ) is a rare, severe debilitating condition from unknown causes. It is characterized by nonhealing exposed bone in a patient with a history of antiresorptive or antiangiogenic agents in the absence of radiation exposure to the head and neck region. The first case of MRONJ was reported in the early 2000s. Diagnostic criteria for MRONJ was developed by the American Association of Oral and Maxillofacial Surgeons (AAOMS) based on pharmacological history as well as clinical and radiographic features. Antiresorptive medications such as bisphosphonate and denosumab are currently considered the treatment of choice in patients with osteoclastic bone disease. These reduce bone turnover and improve bone density, thereby improving bone quality. These agents have also been shown to reduce the risk of osteoporotic fractures due to their potent effect in suppressing osteoclastic activity by slowing the remodeling process and increasing bone density, thereby improving quality of life for most of the patients. Despite the great benefits of bisphosphonates and other antiresorptive medications, osteonecrosis of the jaw (ONJ) due to the effects of these medications in the presence of a local risk factor is a significant drawback. Moreover, antiangiogenic drugs play a major role in developing bone necrosis. They are prescribed in cancer cases to prevent metastasis through the blood and lymph nodes. These drugs interfere with the formation of new blood vessels, resulting in ischemia and eventually ONJ. This risk can be managed by evaluating the route and the duration of administration as such a risk can be considered dose-time dependent. As a preventive measure, dental screening before initiating any type of ONJ-related medications can significantly lower the risk of ONJ. Treatment goals can be achieved through pain and infection control, in addition to the management of bone necrosis and resorption. The aim of this review is to identify all causative agents and summarize the preventive measures, diagnostic criteria, and treatment strategies related to MRONJ.

## Introduction and background

Medication-related osteonecrosis of the jaw (MRONJ) is a rare but severe debilitating condition, the exact cause for which has not yet been determined [1,2]. MRONJ is characterized by nonhealing exposed bone in patients with a history or ongoing use of an antiresorptive or antiangiogenic agent and no history of radiation exposure to the head and neck region [2,3].

The first case of MRONJ was reported by Marx in the early 2000s in a study about nonhealing exposed bone in the maxillofacial region of a patient treated with a bisphosphonate, an antiresorptive medication that affects the dissolution of the mineral content of the bone [4-9]. According to the literature, the occurrence of bone necrosis due to this medication is much greater with IV administration compared to the oral route [5]. The incidence of this bony disease among antiresorptive users ranges from 0.7% to 18% [1].

An association between osteonecrosis of the jaw (ONJ) and medications other than bisphosphonate, such as denosumab and antiangiogenic drugs in the treatment of malignancy, has been found with an increased incidence of bone necrosis being related to these medications [6]. To include all causative medications in the diagnostic discourse related to ONJ, the American Association of Oral and Maxillofacial Surgeons (AAOMS) suggested that the nomenclature be changed from bisphosphonate-related ONJ (BRONJ) to MRONJ [6,7]. This review aims to identify all causative agents and summarize the preventive measures, diagnostic criteria, and treatment strategies pertaining to MRONJ.

## Review

Diagnosis and stages of MRONJ

The diagnostic criteria for MRONJ developed by AAOMS are based on pharmacological history as well as clinical and radiographic features [5-9]. A patient can be diagnosed with MRONJ if both of the following criteria are fulfilled: a history or ongoing treatment with antiangiogenic agents or antiresorptives such as bisphosphonate and denosumab; exposed or nonhealing bone that can be probed through a fistula in the maxillofacial region persisting for more than eight weeks and no history of radiation therapy to the head and neck region or obvious metastatic disease of the jaws [1,2,5,6,8].

MRONJ staging system was developed in 2006 by Ruggiero et al. and subsequently adopted by the AAOMS and updated in 2014 [5,6] (Table 1).

**Table 1:** MRONJ staging system as updated by AAOMS in 2014 IV: intravenous; MRONJ: medication-related osteonecrosis of the jaw; AAOMS: American Association of Oral and Maxillofacial Surgeons

Stage	Clinical findings
At-risk category	No apparent necrotic bone in patients who have been treated with either oral or IV bisphosphonates
Stage 0	No clinical evidence of necrotic bone, but non-specific clinical findings, radiographic changes, and symptoms
Stage 1	Exposed and necrotic bone, or fistulae that probes to the bone in patients who are asymptomatic and have no evidence of infection
Stage 2	Exposed and necrotic bone, or fistulae that probes to the bone, associated with infection as evidenced by pain and erythema in the region of the exposed bone, with or without purulent drainage
Stage 3	Exposed and necrotic bone or a fistula that probes to bone in patients with pain, infection, and one or more of the following: exposed and necrotic bone extending beyond the region of alveolar bone (i.e., inferior border and ramus in the mandible, maxillary sinus, and zygoma in the maxilla) resulting in pathologic fracture, extra-oral fistula, oral-antral/oral-nasal communication or osteolysis extending to the inferior border of the mandible of sinus floor

Stage 0 was added to the updated version by AAOMS, representing the prodromal period and nonspecific clinical or radiographic symptoms before any evidence of bone exposure [6]. These symptoms can manifest clinically as a toothache from a nonodontogenic cause, radiating pain, unexplained pain, or thickening of the sinus wall and altered sensation. Radiographically, it can be an unexplained bone loss not attributed to periodontal inflammation with a change in trabecular bone pattern [6].

Imaging modalities (orthopantomography/CT/MRI)

**Figure 1:**
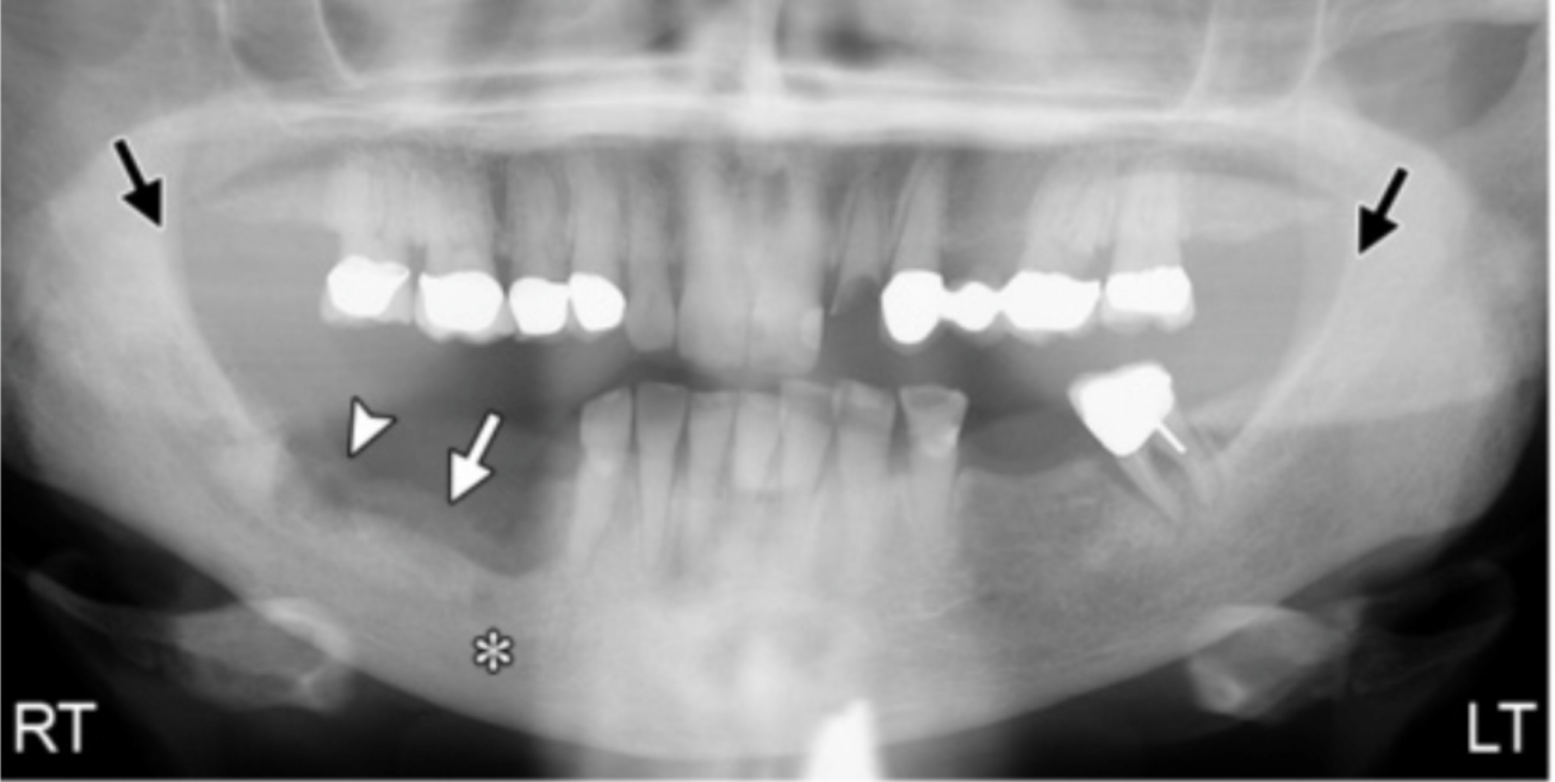
The appearance of ONJ on panoramic radiograph Panoramic radiograph view of ONJ shows alveolar bone reaction at the area of the empty socket of previously extracted teeth (arrowhead and white arrow), and loss of cortication on the right side compared to the left (black arrows) *Sclerosis ONJ: osteonecrosis of the jaw

The panoramic examination provides an overall view to examine the whole mandible and maxilla [10,11]. It can be an unexplained bone loss not attributed to periodontal inflammation with a change in trabecular bone pattern [6,12,13]. Radiographic features can manifest in the early stage through orthopantomography as diffuse sclerotic bone, ill-defined radiolucency, or a mix of a radiopaque and radiolucent lesion in addition to a nonhealing extraction socket [5] (Figure 1).

In advanced stages, the radiographic presentation of MRONJ may mimic the classic appearance of chronic osteomyelitis as sequestrum formation, thickening of lamina dura, and pathological fractures [7]. For a more detailed examination, digital imaging as in CT and cone-beam CT (CBCT) provide high-quality tomographic images to reveal MRONJ/ARONJ lesions [10-12]. Diffuse osteosclerosis, bone resorption, degenerated cortical bone, periosteal reaction, and bone fistulas are findings that reveal the spread and the extent of such a lesion [11].

**Figure 2:**
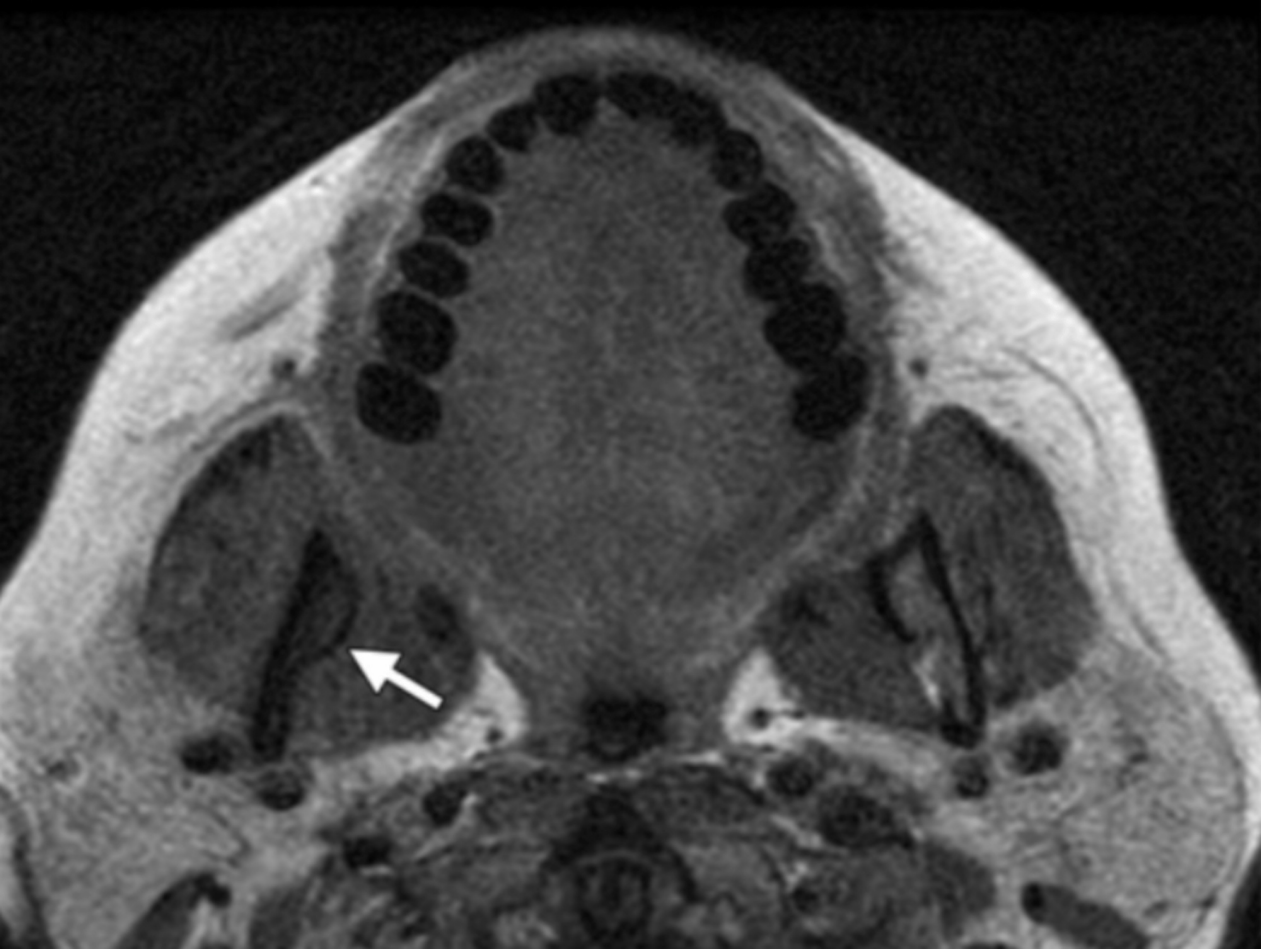
The appearance of ONJ on MRI modality: T1-weighted image The image shows reduced signal intensity in the right mandibular ramus (white arrow) ONJ: osteonecrosis of the jaw; MRI: magnetic resonance imaging

**Figure 3:**
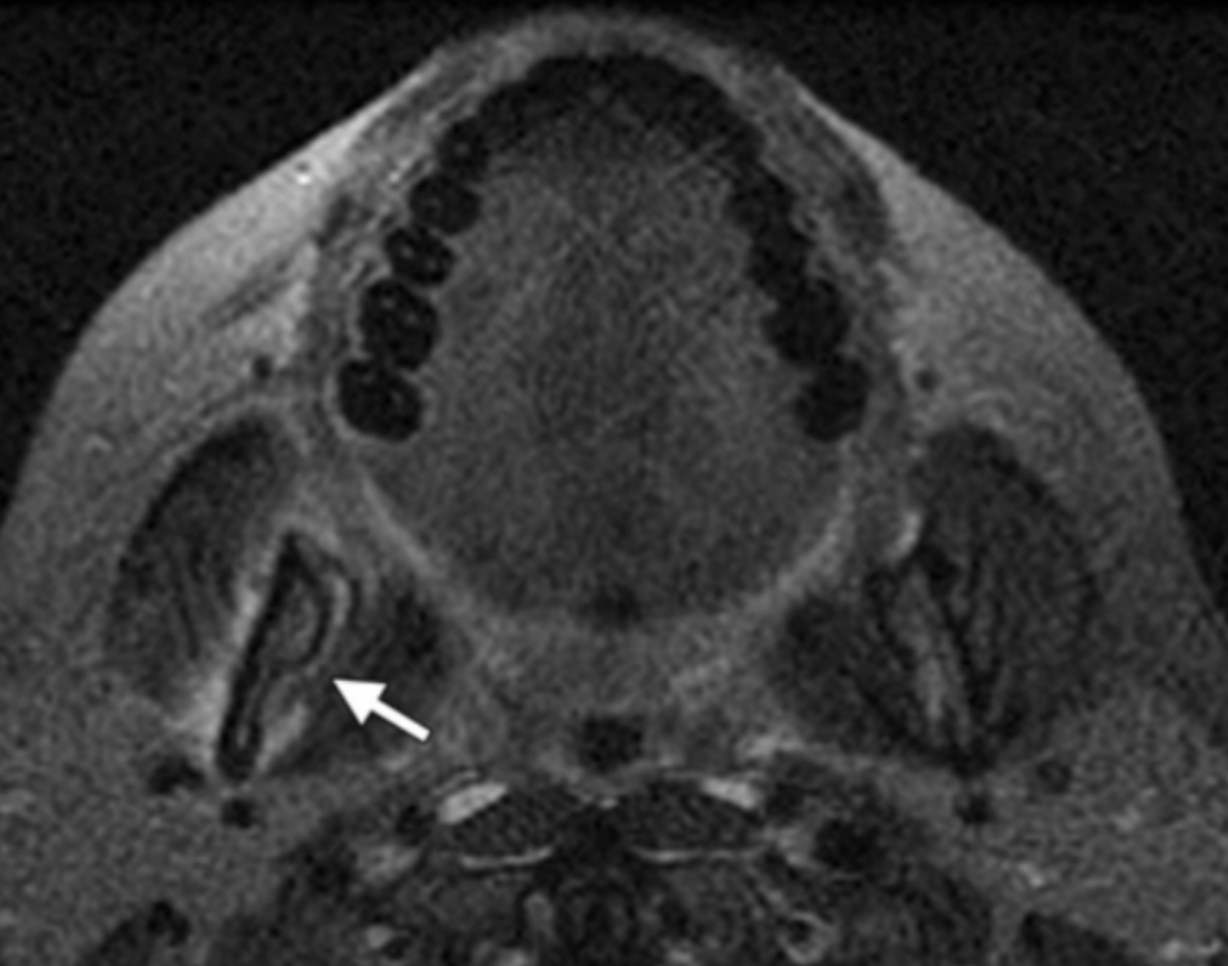
The appearance of ONJ on MRI modality: T2-weighted image Tissue window with increased signal intensity in the adjacent soft tissues of the right mandible (white arrow) ONJ: osteonecrosis of the jaw; MRI: magnetic resonance imaging

CBCT is superior to CT since it exhibits a higher resolution in the alveolar bone and the jawbones. MRI scans are less precise in skeletal imaging than CT scans. Hence, the appearance of ONJ on MRI is variable and unpredictable [11]. The MRI diagnostic modality depends mainly on the signal intensity alteration on the bone and adjacent soft tissues. Studies showed varied signal intensity on T1 and T2, which were believed to be stage-related changes. The T1-weighted image showed reduced signal intensity (Figure 2). The T2-weighted image showed increased signal intensity in the early stage of the disease and increased or decreased signal intensity in advanced stages of the disease (Figure 3). This variability in the T2-weighted image was believed to be due to the nature of the wound and the stage of the disease [13].

 

**Figure 4:**
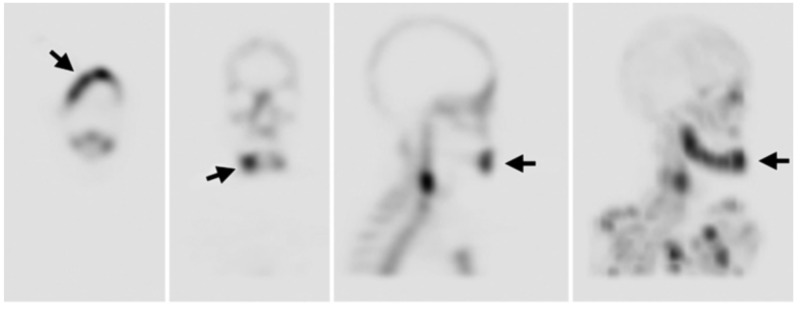
SPECT bone scintigraphy SPECT bone scintigraphy shows increased uptake in the right mandible (black arrows) SPECT: single-photon emission tomography

The diagnostic value of MRI in ONJ not well established. However, the main advantage of MRI over other imaging modalities is the ability to assess the degree of extent of the lesion in bone and soft tissues, which helps in planning for surgical debridement and resection [8]. Diffusion-weighted imaging (DWI) is utilized for treatment response forecasting. DWI is used in monitoring and predicting recurrence of highly recurrent lesions. Because of its short processing time, it could be used along with an MRI protocol [14]. A newly introduced modality called bone scintigraphy provides less information about anatomical configuration than the CT or MRI. For MRONJ patients, single-photon emission computed tomography (SPECT) has been used for localization of physiological changes in the bone [10-12]. It can be used to assess the activity of the surrounding bone (Figure 4) [13]. Despite the advantage of this modality, it appears to be sensitive but not specific [13].

Pathogenesis and mechanism

Various medical conditions can manifest by progressive bone loss via increasing the activity of osteoclasts [9,15-17]. Antiresorptive medications such as bisphosphonate and denosumab are currently considered the treatment of choice in patients with osteoclastic bone disease or tumors that reduce bone turnover as these agents improve bone density and thereby bone quality [16]. These drugs can be prescribed to stop the progression of this effect in cases of osteoporosis and metastatic bone cancers [9,15,16]. Bisphosphonate and denosumab are considered the main antiresorptive drugs and have the most powerful effect. Furthermore, antiangiogenic drugs play a major role in developing bone necrosis [17].

Antiresorptive agents

Bisphosphonate

Bisphosphonate is a well-known antiresorptive medication [16,18]. It acts at a cellular level, targeting osteoclasts and disrupting their function [16,18]. It can be given via different routes according to the potency, either orally or intravenously. Based on the recommendations by the American Society of Clinical Oncology (ASCO), guidelines have been established for bisphosphonate use, and the drug is considered the standard of care in the treatment of hypercalcemia associated with malignancy and metastatic bone lesions associated with multiple myeloma and breast cancer [5,6]. Also, bisphosphonate is currently the treatment of choice for osteoporosis [5,6]. It has been shown to reduce the risk of osteoporotic fractures due to its potent effect in suppressing the osteoclastic activity by slowing the remodeling process and increasing bone density, thereby improving the quality of life in most patients [5]. Despite the great benefits of bisphosphonates and other antiresorptive medications, ONJ that results from the effects of these medications in the presence of a local risk factor is a significant drawback [5,19]. While the mechanism of action of bisphosphonates is not completely understood, multiple studies have suggested that bisphosphonate has a high affinity to hydroxyapatite crystals forming the bone, thereby inhibiting the resorptive action of osteoclasts by induced apoptosis [19-21]. Moreover, it may be indirectly acting on the osteoblasts by preventing differentiation due to the lack of cytokines released from the osteoclast preventing the bone healing ability; this elaborates on the concept of BRONJ [16]. Bisphosphonate therapy can be either non-nitrogen-containing bisphosphonates (etidronate, clodronate, tiludronate) or nitrogen-containing bisphosphonates (pamidronate, alendronate, ibandronate, risedronate, zoledronic acid) [16]. While 95% of the drug will be released outside the body within six hours, the half-life of a given drug may last more than 10 years due to its strong affinity to the bone [19,20]. The bone remodeling process is further regulated by balancing between the receptor activator of nuclear factor-kappa-Β (RANK) ligand (RANKL) cytokine produced by osteoblasts to promote bone resorption or osteoprotegerin (OPG), which inhibit bone resorption process by preventing binding of RANK/RANKL [20].

Denosumab

Denosumab is a newly developed antiresorptive medication. It is an anti-RANKL antibody that has the same mechanism of action as OPG produced by osteoblasts [16]. By blocking RANKL/RANK interaction, it disrupts osteoclast formation, differentiation, and survival, thereby decreasing bone resorption [16]. Denosumab is used in the treatment of osteoporosis and other malignant bone diseases [16,20]. It was reported that the risk of developing denosumab-related ONJ (DRONJ) in patients with osteoporosis is 0.01-0.03%, while in cancer patients, this risk ranges from 1-2% [7]. Unlike bisphosphonate, denosumab has a short half-life as RANKL-inhibitors do not bind to the bone; therefore, their effects on the bone do not last very long and are mostly diminished within six months of treatment cessation [6].

The main difference between BRONJ and DRONJ is the time of occurrence; BRONJ can occur between 33 months (with oral administration) to 48 months (with IV administration) [16]. DRONJ, however, occurs early after administration [8]. Moreover, BRONJ is highly dependent on the route, amount, and duration of treatment [8].

ONJ associated with antiangiogenics

Antiangiogenic drugs are monoclonal antibodies targeting vascular endothelial growth factor (VEGF) receptors [22]. Angiogenesis is the process of blood vessel formation through endothelium cell differentiation. It favorably affects tumor growth and induces tumor invasion of vessels and adjacent lymph, resulting in tumor metastasis. Antiangiogenic medications are prescribed in cancer cases to prevent metastasis through the blood and lymph nodes [19]. These drugs interfere with the formation of new blood vessels, resulting in ischemia and eventually ONJ [7]. ONJ is believed to be avascular necrosis of the jaw and, therefore, correlating angiogenesis inhibitors to ONJ is logical [18]. The most used antiangiogenic drugs are VEGF inhibitors (e.g., bevacizumab) or tyrosine kinase inhibitors (e.g., sunitinib). Studies have supported that denosumab and antiangiogenic drugs with a shortened half-life do not tend to accumulate in the bone as bisphosphonate does upon long-term use [21].

Risk factors

Among all the factors that can contribute to MRONJ development, the main risk factors can be summarized in three main points: a local risk factor, medical illness, and the type of medication [8]. The use of both bisphosphonate and denosumab are considered primary risk factors for developing ONJ, though other medications such as antiangiogenic drugs have been reported in several studies [2,22]. The risk can be assessed by evaluating the route and the duration of administration [5]. These risks may be considered as dose-time dependent, meaning that as the dose increases for a longer period the risk of developing ONJ increases [8]. Pre-existing dental or periodontal infection in patients treated with antiresorptive or antiangiogenic medications is a well-recognized risk factor for developing MRONJ [2]. Infection increases the acidity in the area of infection, leading to suppression of the mechanism of healing, and thereby resulting in bone necrosis [16]. This is due to the jawbone being more susceptible to infections compared to other bones in the body (it is exposed to millions of bacteria in the oral cavity [6]. Furthermore, when comparing anatomic sites most likely to be affected by bone necrosis due to medications, the mandible is more likely to develop osteonecrosis (73%) than the maxilla (22.5%) [6]. Since there is a single blood supply for the mandible, it is more prone to necrosis and infections [6]. Tooth extraction is the most common precipitating factor for developing bone necrosis [8,21,22]. With 52-61% of the patients reporting tooth extraction as the causative factor [6,21].

A systematic review conducted in 2017 reported that renal cancer has the highest associated rate of MRONJ occurrence, while breast, prostate, and multiple myeloma reported it in 65% of cases. This can be explained as resulting from the combined use of both bisphosphonate and antiangiogenic drugs in treatment. However, in nonmalignant cases, osteoporosis was considered the highest associated risk among all medical conditions [22].

Management and prevention

An MRONJ treatment strategy is not yet well established [17]. Therefore, prevention and case selection are fundamental in reducing the risk of bone necrosis of the jaw [9,15-17]. As a preventive measure, dental screening before initiating any type of ONJ-related medications can significantly lower the risk of ONJ [2]. It is the dentist’s responsibility to identify an individual at risk and prevent dental infection through good oral hygiene and regular dental check-ups [2]. Moreover, all patients undergoing antiresorptive or antiangiogenic therapy should undergo dental screening through a clinical and radiographic assessment to eliminate ongoing acute infections and prevent possible future occurrences. Patients should also be informed about the benefits of prophylactic dental care and should be advised to avoid dentoalveolar surgery [2,5,6]. The goal of treatment can be achieved through pain and infection control in addition to the management of bone necrosis and resorption [2,8].

Taking a drug-holiday before any invasive procedure remains a controversial issue [22]. In this case, a drug holiday can be defined as the temporary termination of drug administration before dentoalveolar surgery to minimize the risk of bone necrosis [22].

Patients undergoing surgical extraction or any other dentoalveolar surgery with a history or current bisphosphonate use through an oral route of administration for less than four years with no clinical risk factor have a low risk of developing MRONJ and require no alteration in the planned procedure [6]. However, patients should be informed about the risk of developing ONJ. Their physician should be involved in the decision making and possible dose alteration or drug holiday [6].

Patients on oral bisphosphonate therapy longer than four years, or less than four years in duration but with concomitant use of an antiangiogenic medication or corticosteroids, will experience a synergistic effect of these therapies on their bone [6]. The physician should suggest the discontinuation of bisphosphonate therapy for at least two months before dentoalveolar surgery only if the systemic condition of the patient permits it; the holiday should be continued until osseous healing and full mucosal coverage are achieved [6].

Treatment

The treatment and management protocol for MRONJ is challenging and remains a controversial topic [12,19-21]. However, the treatment protocol is case-dependent and requires treatment according to the condition stage and symptoms [6]. Multiple treatment approaches have been introduced to control ONJ, including conservative treatment, surgical debridement, and resection of the lesions or the use of other adjunctive treatments such as oxygen therapy or, recently, the use of mesenchymal cells to regenerate the damaged bone [19-21]. The use of these adjunctive modalities is not well supported, and additional studies are required to prove their efficacy [6].

Conservative Approach

This approach is reserved for patients in at-risk, early asymptomatic stages. Moreover, conservative treatment can be implemented in patients who cannot undergo surgical treatment, though this approach may only provide temporary relief of symptoms in 70% of the cases and cannot be considered as a success [17].

Conservative treatment includes keeping good oral hygiene, periodic dental visits, chlorhexidine mouthwash, and antibiotic therapy. This can stabilize or minimally improve the condition. Some studies have reported that a higher success rate with promising results could be achieved through a combination of conservative treatment with adjunctive treatments such as hyperbaric oxygen, ozone therapy, or low-intensity laser therapy [23-25].

Surgical Approach

A surgical approach to any necrotic exposed bone may be introduced whenever conservative management has failed. Bone exposure, as defined in stages II and III, requires surgical intervention. The surgical approach can be either conservative or respective surgery alone or with adjunctive treatment [23].

A conservative surgical approach is achieved through debridement of superficial necrotic bone (sequestrectomy) in addition to antibiotics and antiseptic mouth rinses. Conservative surgery may be combined with other treatments such as ozone therapy and leukocyte-platelet rich fibrin (L-PRF). According to Agrillo et al., 60% of cases were healed with ozone therapy, compared with 77% of cases healed when treated with L-PRF according to Kim et al. [23-25].

Segmental resection is applied with advanced cases of MRONJ and when conservative debridement has failed. This is characterized by the removal of all necrotic material, leaving only healthy bone. The challenge in segmental resection is the difficulty in obtaining pure, healthy bone [23-25].

Management approach

According to the Ruggiero classification developed in 2006 and updated by AAOMS in 2014, the treatment approach differs according to the stage and symptoms [6].

At-risk patients with a history of antiresorptive or antiangiogenic drugs require no treatment with close clinical and radiographic monitoring. However, they need to be educated about the possibility of bone exposure and further bone necrosis. They also need to be informed about the possible signs and symptoms of the condition and should be advised to seek dental management as soon as the condition is noticed. Therefore, it is critical that patients be educated about the importance of dental hygiene, regular dental follow-up, and conservative dental treatment that plays a major role in reducing the risk of bone necrosis.

Stages of MRONJ and Treatment Approach

Stage 0: since this stage represents a prodromal period with no specific symptoms, the treatment objective is only symptomatic treatment to control pain and infections, in addition to close monitoring for any sign of progression in the clinical state or radiographic image.

Patients with established ONJ are treated differently; the treatment objectives are mainly focused on controlling pain, infection, and the progression of the bone necrosis.

Stage 1: In this stage, the patient is asymptomatic, but with evidence of bone exposure. The treatment is chlorhexidine mouthwash 0.12% and regular follow-up appointments. Neither antibiotic nor surgical intervention is required in this stage.

Stage 2: In this stage, due to evidence of necrosis and associated infection, an antibiotic regimen with an antimicrobial mouthwash is the treatment of choice.

Stage 3: Surgical management is indicated in combination with an antibiotic regimen in this stage. The surgical approach varies between debridement to complete resection with possible immediate reconstruction with plates or obturators.

## Conclusions

Despite the strong association between jaw necrosis and bisphosphonates and other antiresorptive medications and antiangiogenic drugs, the pathophysiology of MRONJ is not completely understood. Hence, an effective and appropriate therapy for the condition is still to be decided. It is crucial to have a collaborative approach involving dentists, prescribing doctors, and pharmacists to prevent the development of MRONJ.

## References

[1] Peer A, Khamaisi M (2015). Diabetes as a risk factor for medication-related osteonecrosis of the jaw. J Dent Res.

[2] Di Fede O, Panzarella V, Mauceri R (2018). The dental management of patients at risk of medication-related osteonecrosis of the jaw: new paradigm of primary prevention. Biomed Res Int.

[3] Akashi M, Kusumoto J, Takeda D, Shigeta T, Hasegawa T, Komori T (2018). A literature review of perioperative antibiotic administration in surgery for medication-related osteonecrosis of the jaw. Oral Maxillofac Surg.

[4] Marx RE (2003). Pamidronate (Aredia) and zoledronate (Zometa) induced avascular necrosis of the jaws: a growing epidemic. J Oral Maxillofac Surg.

[5] Ruggiero SL (2009). Bisphosphonate-related osteonecrosis of the jaw (BRONJ): initial discovery and subsequent development. J Oral Maxillofac Surg.

[6] Ruggiero SL, Dodson TB, Fantasia J, Goodday R, Aghaloo T, Mehrotra B, O’Ryan F (2014). American Association of Oral and Maxillofacial Surgeons position paper on medication-related osteonecrosis of the jaw—2014 update. J Oral Maxillofac Surg.

[7] Khan AA, Morrison A, Kendler DL (2017). Case-based review of osteonecrosis of the jaw (ONJ) and application of the international recommendations for management from the International Task Force on ONJ. J Clin Densitom.

[8] McGowan K, McGowan T, Ivanovski S (2018). Risk factors for medication-related osteonecrosis of the jaws: a systematic review. Oral Dis.

[9] Lerman MA, Xie W, Treister NS, Richardson PG, Weller EA, Woo SB (2013). Conservative management of bisphosphonate-related osteonecrosis of the jaws: staging and treatment outcomes. Oral Oncol.

[10] Stockmann P, Hinkmann FM, Lell MM, Fenner M, Vairaktaris E, Neukam FW, Nkenke E (2010). Panoramic Radiograph, computed tomography or magnetic resonance imaging. Which imaging technique should be preferred in bisphosphonate-associated osteonecrosis of the jaw? A prospective clinical study. Clin Oral Investig.

[11] Tsuchimochi M, Kurabayashi T (2019). Symposium: imaging modalities for drug-related osteonecrosis of the jaw (1), role of imaging in drug-related osteonecrosis of the jaw: An up-to-date review (secondary publication). Jpn Dent Sci Rev.

[12] Shimamoto H, Grogan TR, Tsujimoto T (2018). Does CBCT alter the diagnostic thinking efficacy, management and prognosis of patients with suspected Stage 0 medication-related osteonecrosis of the jaws?. Dentomaxillofac Radiol.

[13] Morag Y, Morag-Hezroni M, Jamadar DA, Ward BB, Jacobson JA, Zwetchkenbaum SR, Helman J (2009). Bisphosphonate-related osteonecrosis of the jaw: a pictorial review. Radiographics.

[14] Bhatt N, Gupta N, Soni N, Hooda K, Sapire JM, Kumar Y (2017). Role of diffusion-weighted imaging in head and neck lesions: pictorial review. Neuroradiol J.

[15] Voss PJ, Poxleitner P, Schmelzeisen R, Stricker A, Semper-Hogg W (2017). Update MRONJ and perspectives of its treatment. J Stomatol Oral Maxillofac Surg.

[16] Shibahara T (2019). Antiresorptive agent-related osteonecrosis of the jaw (ARONJ): A twist of fate in the bone. Tohoku J Exp Med.

[17] Rosella D, Papi P, Giardino R, Cicalini E, Piccoli L, Pompa G (2016). Medication-related osteonecrosis of the jaw: clinical and practical guidelines. J Int Soc Prev Community Dent.

[18] Aljohani S, Fliefel R, Ihbe J, Kühnisch J, Ehrenfeld M, Otto S (2017). What is the effect of anti-resorptive drugs (ARDs) on the development of medication-related osteonecrosis of the jaw (MRONJ) in osteoporosis patients: a systematic review. J Craniomaxillofac Surg.

[19] Lombard T, Neirinckx V, Rogister B, Gilon Y, Wislet S (2016). Medication-related osteonecrosis of the jaw: new insights into molecular mechanisms and cellular therapeutic approaches. Stem Cells Int.

[20] Favia G, Tempesta A, Limongelli L, Crincoli V, Maiorano E (2018). Medication-related osteonecrosis of the jaw: surgical or non-surgical treatment?. Oral Dis.

[21] Kuroshima S, Sasaki M, Murata H, Sawase T (2019). Medication‐related osteonecrosis of the jaw‐like lesions in rodents: a comprehensive systematic review and meta‐analysis. Gerodontology.

[22] Kuroshima S, Sasaki M, Sawase T (2019). Medication-related osteonecrosis of the jaw: a literature review. J Oral Biosci.

[23] Agrillo A, Filiaci F, Ramieri V (2012). Bisphosphonate-related osteonecrosis of the jaw (BRONJ): 5 year experience in the treatment of 131 cases with ozone therapy. Eur Rev Med Pharmacol Sci.

[24] Kim JW, Kim SJ, Kim MR (2014). Leucocyte-rich and platelet-rich fibrin for the treatment of bisphosphonate-related osteonecrosis of the jaw: a prospective feasibility study. Br J Oral Maxillofac Surg.

[25] Rodriguez-Lozano FJ, Oñate-Sanchez RE (2016). Treatment of osteonecrosis of the jaw related to bisphosphonates and other antiresorptive agents. Med Oral Patol Oral Cir Bucal.

